# Race and ethnicity in physiological research: When socio‐political constructs and biology collide

**DOI:** 10.1113/EP091409

**Published:** 2024-05-03

**Authors:** Ronan M. G. Berg, Damian M. Bailey

**Affiliations:** ^1^ Centre for Physical Activity Research Copenhagen University Hospital – Rigshospitalet Copenhagen Denmark; ^2^ Department of Clinical Physiology and Nuclear Medicine Copenhagen University Hospital – Rigshospitalet Copenhagen Denmark; ^3^ Neurovascular Research Laboratory, Faculty of Life Sciences and Education University of South Wales Pontypridd UK; ^4^ Department of Biomedical Sciences, Faculty of Health and Medical Sciences University of Copenhagen Copenhagen Denmark

In biological taxonomy, the term *species* refers to a group of similar organisms that can only reproduce with each other, while the term *race* lacks a consistent definition. Genetically and biologically, *Homo sapiens* is a single species with genetic diversity (Yudell et al., 2016), and while the concept of different human races does have biological connotations, it is a construct based on region‐specific cultural and historical ideas of how groups can be delineated by differences in phenotype, skin colour, ancestry, socioeconomic status and geographical location, while ethnicity is a related concept linked with cultural expression and identification (Mohsen, 2020). To this day, race and ethnicity corrections do exist in reference materials for several clinical physiological metrics, such as lung volumes, kidney function and bone mineral density, despite their lack of biological foundation (Crandall et al., 2023; Elmaleh‐Sachs et al., 2022; Tsai et al., 2021).

The concept of race was prevalent in European and American academic writings from the 1700s to the end of World War II. Early attempts to classify human populations by Carl Linnaeus (1707–1778) and Johann Friedrich Blumenbach (1752–1840) resulted in racial categorizations based on physical features and geographic origins. These classifications were influenced by biased narratives, myths and societal prejudices, and served as a vehicle for colonization, enslavement, subordination, marginalization, and genocide in the centuries to follow (Braun et al., 2005; Mohsen, 2020). However, scientific advancements in the 20th century revealed that human genetic variation does not align with a racial model at all. *Homo sapiens* arose in eastern Africa more than 230,000 years ago (Vidal et al., 2022), and all current members of this species share more than 99% of their genetic material, within which the genetic diversity is a result of gradual variation and isolation by distance and time, rather than discrete racial or ethnic categories (Yudell et al., 2016).

A conundrum may arise when considering potential differences in physiological measurements, as well as disease prevalence, presentation and prognosis between racial and ethnic categories (Burchard et al., 2003). However, given the biological basis of the categorization is flawed, this points towards socially mediated mechanisms and environmental factors, conceivably in the form of residual confounding, rather than biology per se (Braun et al., 2005; Lujan & DiCarlo, 2021). In fact, the use of race‐corrections in clinical physiological metrics has been shown to contribute to, rather than prevent, misclassification of disease and thus to enforce health inequalities (Ahmed et al., 2021; Bhakta et al., 2023; Moffett et al., 2023; Tsai et al., 2021). Recent efforts to establish reference values for lung and kidney function based on diverse populations, without race‐corrections, have in many cases led to more accurate diagnostic classifications (Bhakta et al., 2023; Delgado et al., 2022; Elmaleh‐Sachs et al., 2022; Raynaud et al., 2023), thus providing definitive arguments against race‐corrections of these physiological metrics.

Although the concepts of race and ethnicity hold no biological validity, their social implications are profound. They continue to shape the distribution of wealth, power dynamics, and opportunities worldwide. This perpetuates social exclusion, discrimination and violence against specific social groups while granting social privileges to others, leading to disparities in both social and physical aspects, and ultimately affecting the physiology of the human body. Consequently, dismissing the reporting of race and ethnicity in scientifically relevant contexts would seem inappropriate (Borrell et al., 2021). In fact, doing so could worsen health inequities, as these categories may provide crucial insights into the social and environmental determinants of health, including discrimination and socioeconomic status. However, it is crucial to recognize that although potential physiological differences may be documented between groups classified by race (or ethnicity), this does not imply that human race is a genetic or biological entity.

## AUTHOR CONTRIBUTIONS

Ronan M. G. Berg: conception, first draft, revisions. Damian M. Bailey: conception, revisions. All authors approved the final version of the manuscript and agree to be accountable for all aspects of the work in ensuring that questions related to the accuracy or integrity of any part of the work are appropriately investigated and resolved. Ronan M. G. Berg is guarantor of this work and accepts full responsibility for the work, and controlled the decision to publish.

## CONFLICT OF INTEREST

The authors declare no conflicts of interest.

## FUNDING INFORMATION

The Centre for Physical Activity Research is supported by TrygFonden Grants ID 101390, ID 20045, and ID 125132. D.M.B. has received funding from a Royal Society Wolfson Research Fellowship (#WM170007) and the Higher Education Funding Council for Wales.
